# PReS-FINAL-2211: NOD2/CARD15 polymorphisms and clinical features in patients with non-infectious uveitis

**DOI:** 10.1186/1546-0096-11-S2-P201

**Published:** 2013-12-05

**Authors:** E Marrani, R Cimaz, OM Lucherini, R Caputo, A Vitale, L Cantarini, G Simonini

**Affiliations:** 1 Pediatric Rheumatology, "Anna Meyer" Children Hospital, Florence, Italy; 2 Rheumatology, "Santa Maria alle Scotte" Hospital, Siena, Italy; 3 Pediatric Ophthalmology, "Anna Meyer" Children Hospital, Florence, Italy

## Introduction

Non-infectious uveitis represents an heterogeneous group of immune-mediated disorders affecting both the uveal tract and the adjacent structures. These diseases are important in clinical practice because they represent one of the most common cause of blindness even in the pediatric age and often require iimmunosuppressive therapy and a multidisciplinary approach. The aetiology of these inflammatory conditions remains unknown. Mutations affecting NOD2/CARD15 gene are responsible for a rare autosomal-dominant disorder, Blau Syndrome, which is characterized by the triad of granulomatous arthritis, skin rashes and uveitis.

## Objectives

Aim of our study is to assess if NOD2-polymorphisms or mutations have a role in the etiology or in the clinical course of patients with non infectious uveitis, either idiopathic or associated with other inflammatory diseases.

## Methods

We enrolled 18 patients: 12 pediatric patients followed at the pediatric hospital "Anna Meyer" (Florence, Italy) and 6 patients (5 adults and 1 child) followed at the Hospital "Santa Maria alle Scotte" (Siena, Italy). Data regarding age of onset, type and localization of uveitis, associated diseases, relapse frequency and complications were collected and recorded with a customized database. NOD2 gene was genotyped in all cases. A statistical analysis has been carried out in order to assess the relationship between NOD2 variations and phenotype.

## Results

NOD2/CARD15 gene variants have been identified in 12 patients: 9 patients showed the polymorphism P268S/SNP5 as heterozygous carriers while 2 patients were homozygous for the same polymorphism. One patient carried two different mutations: the polymorphism P268S/SNP5 in heterozygous and a mutation on intron 3 (c647 18-16 TCT).

We tried to assess if the two genetic variants, identified in our cohort of patients, could impact the clinical course of the disease but we failed to show any associations.

## Conclusion

Our preliminary results suggest that NOD2 variants, except for the Blau related ones, are not implicated in the pathogenesis of uveitis, either idiophatic or associated with other diseases. To our knowledge, this is the first report on the relationship between NOD2 gene and the phenotype of patients affected by uveitis.

## Disclosure of interest

None declared.

